# The relationship between perianal fistula activity and abdominal adipose tissue in Crohn’s disease: an observational study

**DOI:** 10.1186/s13244-022-01293-6

**Published:** 2022-09-24

**Authors:** Ziman Xiong, Ziling Zhou, Luwen Hao, Yuanqiu Li, Xuemei Hu, Daoyu Hu, Yan Luo, Yanchun Wang, Yaqi Shen, Zhen Li

**Affiliations:** 1 Department of Radiology, Tongji Hospital, Tongji Medical College, Huazhong University of Science and Technology, 1095 Jiefang Avenue, Qiaokou District, Wuhan, 430030 Hubei China; 2 Biomedical Engineering Department, College of Life Sciences and Technology, Huazhong University of Science and Technology, Wuhan, China

**Keywords:** Crohn's disease, Perianal fistula, Visceral adipose tissue, Tomography (X-ray computed), Magnetic resonance imaging

## Abstract

**Objective:**

The aim of this study was to analyze the relationship between abdominal adipose tissue and perianal fistula activity in patients with Crohn's disease (CD) using cross-sectional imaging.

**Methods:**

Patients with perianal fistulizing CD who underwent pelvic magnetic resonance imaging (MRI) and abdominal computed tomography (CT) were retrospectively enrolled. We scored the fistulas in each patient's MRI images based on Van Assche's classification. The area and density of subcutaneous adipose tissue (SAT) and visceral adipose tissue (VAT) (at the third, fourth, and fifth lumbar (L3, L4, and L5) levels were measured by averaging five slices of measurements at each vertebral level in CT images, and areas were further standardized by the lumbar height^2^ (height_L1-5_). The VAT/SAT ratio (VSR) and VAT/Total adipose tissue (VA/TA) index were calculated. Based on MRI scores, patients were divided into two groups with low and high activity, and their clinical, MRI features, and CT parameters were compared. We evaluated patients with follow-up MRI and compared the differences in clinical and radiological indicators among patients with different outcomes.

**Results:**

Overall, 136 patients were included, 77 in the low-activity group and 59 in the high-activity group. Patients in the high activity group had lower subcutaneous adipose index (all levels, *p* < 0.05) and visceral adipose index (L3 level, *p* < 0.01), but higher SAT and VAT density (all levels, *p* < 0.01), VSR (L5 level, *p* = 0.07) and VA/TA index (L5 level, *p* < 0.05).

**Conclusion:**

There were differences in adipose tissue composition among CD patients with different active perianal fistulas.

**Supplementary Information:**

The online version contains supplementary material available at 10.1186/s13244-022-01293-6.

## Key points

- Pelvic MRI is a useful tool for evaluating perianal fistulas.
- Lower VAT density and VA/TA index correspond to hypoactive perianal fistulas.
- Perianal fistula activity in CD patients is associated with overall inflammatory load.

## Background

Perianal fistulas are common in the course of Crohn's disease (CD), with anorectal fistulas reported to occur in 20–30% of patients [1–3]. Different from the perianal fistula in general patients caused by anal gland infection, lesions in CD patients are a kind of penetrating inflammation [4], often with a complex course, and therefore requires multidisciplinary management [5].Pelvic magnetic resonance imaging (MRI) and endoscopic ultrasound are accurate in the evaluation of perianal disease in CD patients [6, 7], especially the former, which has been proved by Buchanan et al. to be more accurate than transrectal ultrasonography (TRUS) and rectal palpation in detecting abscesses and fistula classification [8]. In addition, MRI is more suitable for patients with significant pain than TRUS, which is invasive. The Van Assche classification based on MRI is a commonly used method to evaluate the activity of perianal fistula, which combines the anatomy and inflammation to give a total score that reflects the severity of the fistula [9]. To date, several studies have used it to monitor the response to treatment of perianal fistulas [10, 11], and modified Van Assche scores have also been developed based on it [12–14].

The role of visceral adipose tissue (VAT) in colonic inflammation has been reported [15], and its abnormal accumulation is often associated with a more severe disease phenotype as well as a poor prognosis in CD patients [16–18]. Computed tomography (CT)-based body composition analysis is currently the gold standard for quantitative assessment of human skeletal muscle and adipose tissue [19]. In addition, the combination of anatomical features in the images allows researchers to quantify the same tissue in different regions separately, for example subcutaneous adipose tissue (SAT) and VAT. Although there is a growing number of studies using CT-based fat parameters for the analysis of disease activity and prognosis in CD patients [20–22], no researcher has confirmed the difference in these parameters among patients with different perianal fistula activity. In addition, no clear evidence could be found to confirm the relationship between perianal fistula and overall disease activity. Therefore, the main purpose of this study was to investigate the relationship between perianal fistula activity and adipose tissue features as well as disease activity of CD patients by analyzing their CT body composition parameters and fistula on pelvic MRI.

## Methods

### Study participants

The local review board approved this retrospective study, and the requirement of informed consent was waived. Patients diagnosed with Crohn's disease between January 2014 and August 2021 at our institution and with signs of perianal fistula on pelvic MRI images were enrolled. The diagnosis was based on the most recent guidelines of the time [7, 23, 24], and the sign of perianal fistula on pelvic MRI images is a high signal track on T2-weighted images, communicating with the internal orifice of the anorectum [25].

Inclusion criteria were as follows: (a) pelvic MRI and abdominal CT were performed during the same period (within 2 weeks); (b) clinical data and images were available. Exclusion criteria were as follows: (a) the diagnosis of perianal fistula was excluded after review by a senior radiologist; (b) no contemporaneous abdominal CT scan; (c) a bowel resection was received prior to the abdominal CT scan (except appendectomy); (d) clinical data or images were missing. We recorded clinical information of all enrolled patients, including age, gender, clinical symptoms, Montreal classification of CD [26], perianal disease activity index (PDAI) [27] and baseline laboratory parameters (C-reactive protein, CRP and erythrocyte sedimentation rate, ESR). For patients who had undergone perianal fistula surgery, in addition to recording the time of CD diagnosis, we also recorded the time of their initial surgery. For those of these patients who had a follow-up pelvic MRI, we recorded the time of their follow-up and the treatment they received during this period.

### MRI evaluation

Pelvic MRI was performed on one of three 3.0 T machines (GE Discovery 750HD (1 set), GE Healthcare; Siemens MAGNETOM Skyra (2 sets), Siemens AG) with a pelvic phased-array coil. The scan area includes the entire perineum, pelvic floor, and lower pelvic cavity. The following images should be obtained from the scan: sagittal T2-weighted fast spin echo (FSE) images, oblique axial T2-weighted FSE, diffusion-weighted, and T1-weighted FSE images perpendicular to the long axis of the anal canal, and coronal T2-weighted FSE images parallel to the long axis of the anal canal. Gadolinium-based enhanced T1-weighted imaging is not included in our routine scan. Baseline pelvic MRI images of all patients were evaluated separately by two abdominal radiologists (with three and five years of experience in abdominal radiology, respectively) according to the Van Assche's classification [9], with items where the two disagreed being judged by a third radiologist (with eleven years of experience in abdominal radiology). The scores for each item were added to obtain the total MRI score for each patient. After obtaining the MRI scores of all patients, we divided them into two groups of low and high activity. Since the scores for simple fistulas range from 7 to 14, and some fistulas with slightly more complex anatomy but without abscess formation also fell within this range, our grouping rules were as follows: low activity group: score ≤ 14, and no abscess formation; high activity group: score > 14.

### CT analysis

CT images with coverage of the abdomen and pelvis were stored and transmitted in Digital Imaging and Communications in Medicine format for body composition analysis. The distance from the upper edge of the first lumbar vertebra (L1) to the lower edge of the fifth lumbar vertebra (L5) was measured and denoted as height_L1-5_ (Additional file 1: Fig. S1). SAT and VAT were segmented using a semi-automated method, and detailed steps are described in Additional file 1: Supplemental methods. The average cross-sectional area and average density (Hounsfield unit, HU) of SAT and VAT were measured, respectively. The standardized indices subcutaneous adipose index (SAI) and visceral adipose index (VAI) were obtained by dividing the area of SAT and VAT by height_L1-5_ squared [22]. The VAT/SAT ratio (VSR) and VAT/Total adipose tissue (VA/TA) index were also calculated to reflect the proportion of VAT.

### Follow-up evaluation

We evaluated patients for whom follow-up information was available by comparing their follow-up MRI images with baseline MRI images and deriving four outcomes: healed, partial response, unchanged and deterioration according to Ng et al. [11]. For patients who showed low activity at baseline, we classified those who showed healed and partial respond as the responders group and those who remained unchanged and deteriorated as the non-responders group; for patients who showed high activity at baseline, we classified those who remained in high activity as the ‘remained high activity’ group and others as the ‘reduced to low activity’ group, as some patients showed improvement but remained in high activity.

### Statistical analysis

Statistical analysis was carried out through SPSS 26.0 and http://www.statstodo.com/ResourceIndex_Subjects.php website. Quantitative and categorical variables were described as mean ± standard deviation (SD) and frequencies (%), respectively. The agreement between the two radiologists on each MRI assessment was evaluated using the Kappa coefficient, with a Kappa coefficient greater than 0.75 indicating good agreement. Comparisons were made between the high and low activity groups, between the responders and non-responders groups, and between the ‘remained high activity’ and ‘reduced to low activity’ groups. For continuous variables, independent samples *t* test or Mann–Whitney *U* test was performed as appropriate; categorical variables were compared through Chi-squared or Fisher's exact tests; Pearson/Spearman correlation analysis was used for the same CT parameters between different vertebral levels (0.3 ≤ |*r*| < 0.7, moderate; |*r*| > 0.7, high); two-tailed *p* values < 0.05 were considered significant.

## Results

**Fig. 1:**
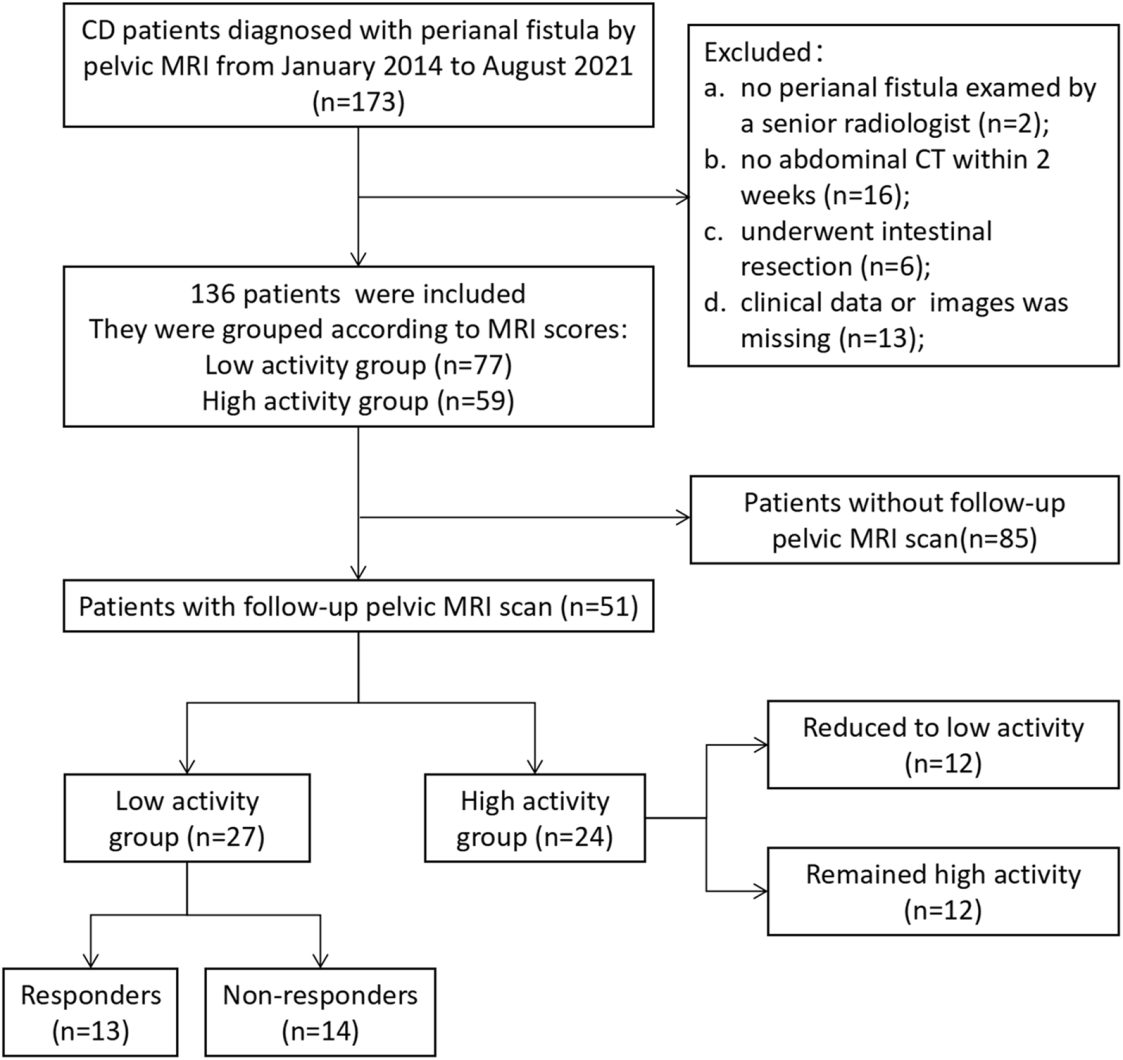
Flowchart for inclusion of patients with Crohn’s disease

A total of 136 CD patients were eventually included in this study, and based on the MRI score of 14, we divided them into two groups, including 77 in the low activity group and 59 in the high activity group (Fig. 1). Of the 47 patients who had received previous perianal fistula-related surgery, 45 (95.7%) underwent surgery prior to being diagnosed with CD, with a median time interval of 24 months. In addition, 64.7% (88/136) of the patients did not present with any perianal fistula-related clinical symptoms.

### Comparison of patients in the low- and high-activity groups

The comparison of clinical indicators between the two groups is shown in Table 1. Both groups were predominantly young males. Patients in the high activity group complained of a higher proportion of clinical symptoms associated with perianal fistula (50.8% vs. 23.4%, *p* = 0.002), and the symptoms would be more complex. In terms of disease behaviors of CD, patients in the high activity group had a higher proportion of stricturing behavior (20.3%), but a low proportion of penetrating lesions (3.4%). In addition, CRP, ESR, and PDAI were higher in the high activity group on admission.

**Table 1:** Demographic data of all patients

	All (*n* = 136)	Low-activity group (*n* = 77)	High-activity group (*n* = 59)	*p* value
Age, years	27.5 ± 10.2	28.7 ± 10.9	25.8 ± 9.0	0.16
Sex				0.45
Male	110	64	46	
Female	26	13	13	
Symptoms (*n*)				**0.002**
Asymptomatic	88 (64.7)	59 (76.6)	29 (49.2)	
Symptomatic	48 (35.3)	18 (23.4)	30 (50.8)	
Pain	16	6	10	
Discharge	10	6	4	
Pain + discharge	22	6	16	
Previous perianal surgery (*n*, %)	47 (34.6)	28 (36.4)	19 (32.2)	0.75
Time from the first surgery to diagnosis, months	*n* = 47	*n* = 28	*n* = 19	0.19
Median (IQR)	24 (5, 36)	24 (6.25, 48)	12 (4, 36)	
Montreal classification (*n*)				
Age				0.46
A1	15	7	8	
A2	105	59	46	
A3	16	11	5	
Location				0.94
L1	29	17	12	
L1 + 4	2	1	1	
L2	34	16	18	
L2 + 4	2	–	2	
L3	57	36	21	
L3 + 4	12	7	5	
Behavior				**0.049**
B1	100	55	45	
B2	21	9	12	
B3	13	11	2	
B2 + 3	2	2	–	
C-reaction protein, mg/L	37.06 ± 33.75 (*n* = 131)	30.81 ± 30.12 (*n* = 75)	45.43 ± 36.70 (*n* = 56)	**0.006**
ESR, mm/h	28.55 ± 21.09 (*n* = 130)	24.08 ± 18.73 (*n* = 75)	34.64 ± 22.73 (*n* = 55)	**0.004**
PDAI (mean ± SD)	3.83 ± 3.41	2.7 ± 1.8	5.3 ± 4.4	** < 0.001**

IQR, interquartile range; SD, standard deviation; ESR, erythrocyte sedimentation rate; PDAI, Perianal Disease Activity Index. Bold values indicate a statistically significant difference (*p* < 0.05)

**Fig. 2:**
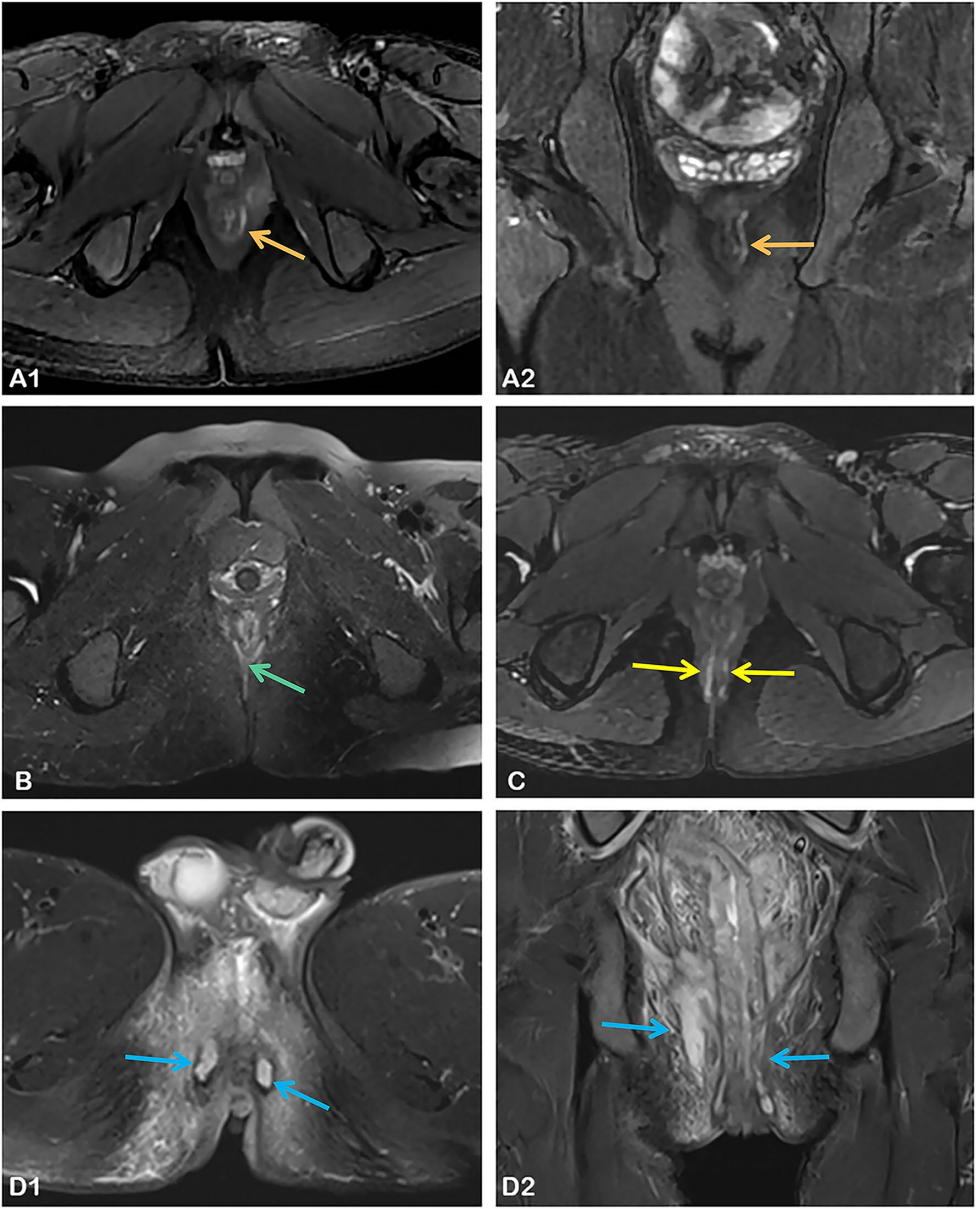
Pelvic MRI images from four patients with Crohn’s disease. **A1**, **A2** Axial and coronal T2-weighted images of an 18-year-old male with linear high-signal fistula between the internal and external anal sphincters without branching or abscess formation (11 points, low activity group#); **B** Axial T2-weighted image of a 31-year-old female with a Y-shaped branching high-signal fistula (12 points, low activity group#); **C** Axial T2-weighted image of a 34-year-old male with multiple high-signal fistula (18 points, high activity group#); **D1**, **D2** Axial and coronal T2-weighted images of a 28-year-old male with multiple perianal abscesses and lesions involving the levator ani muscle (22 points, high activity group#). #Scores were calculated according to the Van Assche’s classification, with 14 or less in the low activity group and the rest in the high activity group

Comparisons of perianal fistula features on MRI images between the two groups are shown in Table 2. Statistical differences were observed between the two groups for each indicators assessed, and patients in the high activity group had higher Van Assche scores. Figure 2 shows MRI images of four patients with different scores. In addition, the Kappa coefficients between the two radiologists were above 0.80 for all assessments (see Additional file 1: Table S1 for specific data), and they were even more in agreement in assessing the number and extension of fistulas, the presence of abscesses, and rectal wall involvement.

**Table 2:** Baseline MRI features of perianal fistula in both groups

	Total (*n* = 136)	Low activity group (*n* = 77)	High activity group (*n* = 59)	*p* value
No. of fistula tracks				< 0.001
Single, unbranched, 1	78	53	25	
Single, branched, 2	17	11	6	
Multiple, 3	41	13	28	
Location				0.000
Extra- or intersphincteric, 1	105	68	37	
Transphincteric, 2	20	9	11	
Suprasphincteric, 3	11	0	11	
Extension				< 0.001
Infralevatoric, 1	125	77	48	
Supralevatoric, 2	11	0	11	
Hyperintensity on T2WI				< 0.001
Mild, 4	24	24	0	
Pronounced, 8	112	53	59	
Collections (cavities > 3 mm diameter)				< 0.001
Absent, 0	84	77	7	
Present, 4	52	0	52	
Rectal wall involvement				0.006
Normal, 0	89	58	31	
Thickened, 2	47	19	28	
Total score				
Mean ± SD	13.6 ± 3.8	10.8 ± 2.1	17.3 ± 2.1	< 0.001
Range	7–22	7–14	15–22	
Fistula type				< 0.001
Simple/complex	53/83	53/24	0/59	

SD, standard deviation

**Fig. 3:**
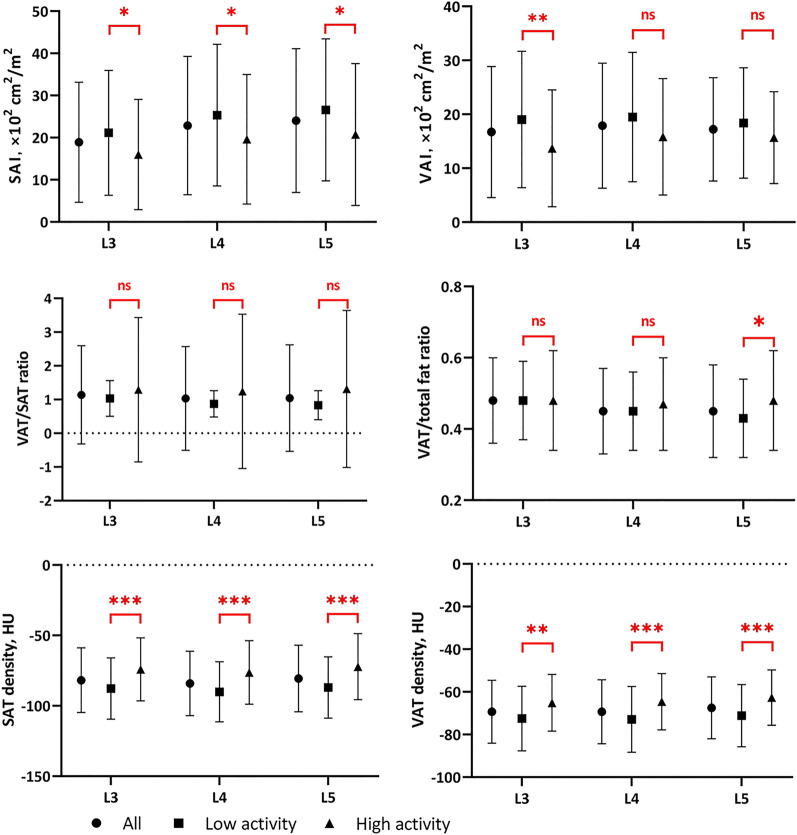
Comparison of CT body composition parameters between low and high activity groups. The first row shows the comparison of SAI and VAI between the two groups, where differences in SAI were observed from L3 to L5 (*p* = 0.015, 0.019, and 0.017, respectively), while differences in VAI were only significant at the L3 level (*p* = 0.008); the second row shows a comparison of the relative content of VAT, where the VSR was not statistically different between the two groups, but we observed a difference in the VA/TA index at the L5 level (*p* = 0.04); the third row shows the comparison between SAT and VAT density, the results showed significant differences in SAT and VAT density between the two groups at all three levels. ns, no significant; *p < 0.05; **p < 0.01; ***p < 0.001. SAT, subcutaneous adipose tissue; VAT, visceral adipose tissue; SAI, subcutaneous adipose index; VAI, visceral adipose index; VSR, VAT/SAT ratio; VA/TA, VAT/Total adipose tissue

The comparison of CT body composition parameters between the two groups is shown in Fig. 3, with specific data available in the supplementary material (Additional file 1: Table S2). CT analysis revealed differences in SAT and VAT parameters between patients with different activity. We found that at all evaluated levels, the low activity group had higher SAI and lower SAT and VAT density. However, higher VAI in the low activity group was only observed at the L3 level. Although statistical differences in VSR were not observed in this study, present results showed that patients in the high activity group had a relatively higher VA/TA index at the L5 level (*p* = 0.04).

### Correlation between CT body composition parameters at different vertebral levels

Our results showed high correlations between the same parameters at the third, fourth and fifth lumbar levels, with specific correlation coefficients shown in Table 3. In addition, for each parameter, correlations are slightly higher between adjacent vertebrae than between spaced vertebrae; and correlations are slightly lower for the VSR and VA/TA indices at all levels compared to the other parameters, but they were still greater than 0.7.

**Table 3:** Pearson/Spearman correlation coefficients between body composition parameters at different vertebral levels

	SAI	VAI	VSR	VA/TA index	SAT density	VAT density
The L3/L4	0.985**	0.958**	0.841**	0.878**	0.983**	0.965**
The L3/L5	0.972**	0.897**	0.708**	0.755**	0.978**	0.937**
The L4/L5	0.979**	0.956**	0.864**	0.882**	0.981**	0.965**

SAI, subcutaneous adipose index; VAI, visceral adipose index; VSR, VAT/SAT ratio; VA/TA: VAT/Total adipose tissue; SAT, subcutaneous adipose tissue; VAT, visceral adipose tissue; L3, third lumbar vertebra; L4, fourth lumbar vertebra; L5, fifth lumbar vertebra

**p < 0.01

### Assessment of follow-up information

**Fig. 4:**
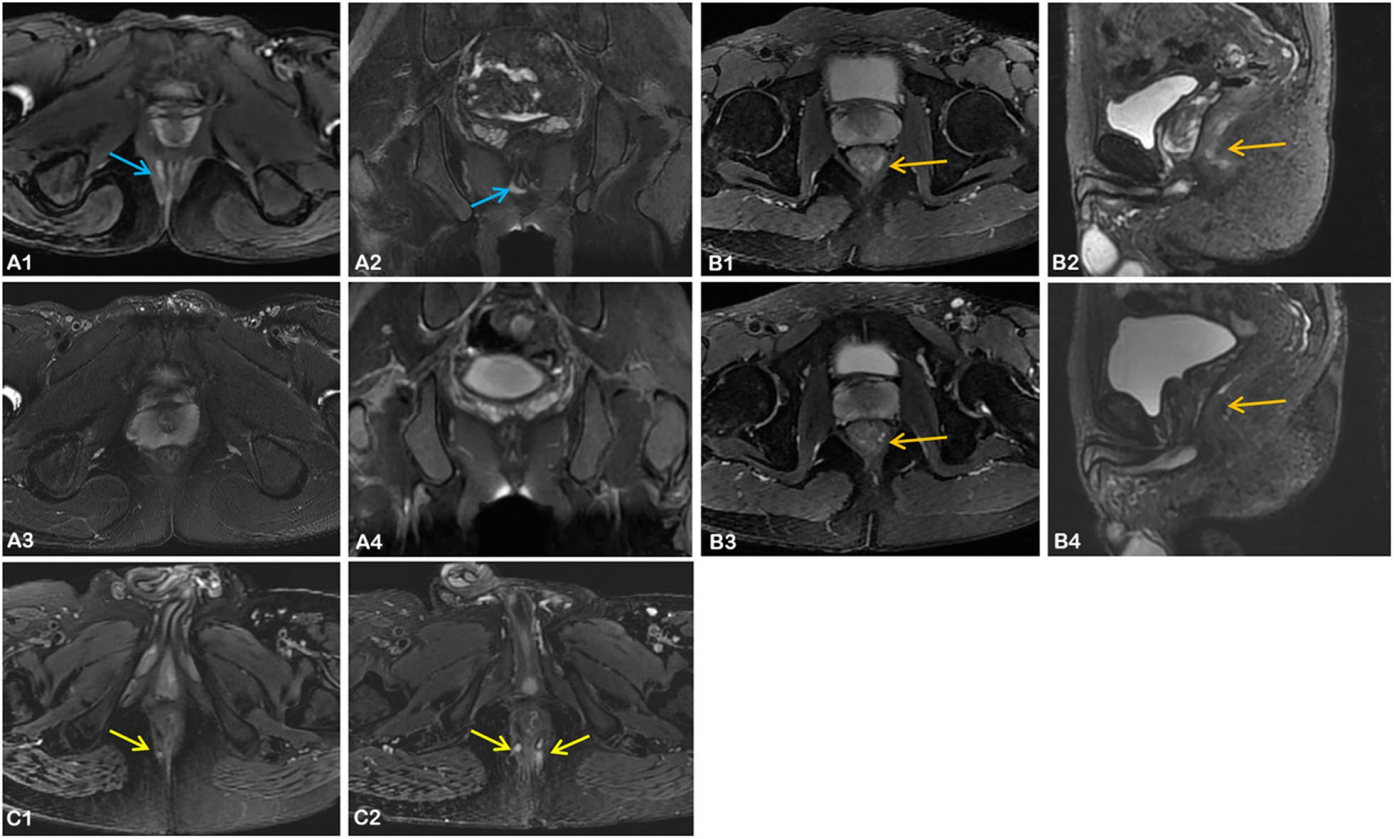
Comparison of baseline and follow-up MRI in three patients with Crohn’s disease. **A1**–**A4** Axial and coronal T2-weighted images of a 22-year-old male from the high activity group, in which a high-signal perianal fistula is visible in baseline images (**A1**, **A2**), and after 15 months of treatment with adalimumab, the corresponding lesion disappeared in follow-up images (**A3**, **A4**); **B1**–**B4** Axial and sagittal T2-weighted images of a 29-year-old male from the low-activity group, where the high T2 signal lesion in baseline images (**B1**, **B2**) was significantly reduced in extent in follow-up images (**B3**, **B4**) after 3 months of treatment with vedolizumab; **C1**, **C2** Axial T2-weighted images of a 34-year-old male from the low-activity group, where a high T2 signal fistula in baseline image (**C1**) became significantly more extensive in follow-up image (**C2**) and an additional lesion was present

Of the 51 patients with follow-up information, 27 were at low activity and 24 were at high activity based on their baseline MRI scores (Table 4). The median follow-up time was about 6 months, but treatment differed between the two groups. Nine patients in the high activity group underwent surgery, whereas more patients in the low activity group received drug therapy (including biological agents, immunosuppressants, Glucocorticoid and 5-Aminosalicylic acid) and only one underwent surgery. After evaluating the follow-up MRI images, we found that 95.8% (23/24) of patients in the high activity group showed changes in their fistulas, with two showing deterioration, twenty-one showing varying degrees of improvement and even four healed, that is, no obvious high signal of perianal fistula was seen on T2WI images (Fig. 4). However, healed was only observed in one patient from the low activity group, and the majority (48.1%, 13/27) showed no significant change. In the follow-up analysis (Additional file 1: Tables S3 and S4), patients with low PDAI responded better regardless of baseline status. Differences in CT parameters between different short-term outcomes were not observed.

**Table 4:** Follow-up information of fifty-one patients

	Total (*n* = 51)	Low activity group (*n* = 27)	High activity group (*n* = 24)	*p* value
Time interval, months				
Median (IQR)	6 (4, 15)	6 (4, 11)	6.5 (4, 17.25)	0.78
Therapy				**0.02**
Drugs	34	22	12	
Surgery	3	0	3	
Drugs + surgery	7	1	6	
Untreated	7	4	3	
Ng score				**0.001**
Healed	5	1 (3.7)	4 (16.7)	
Partial response	29	12 (44.4)	17 (70.8)	
Unchanged	14	13 (48.1)	1 (4.2)	
Deterioration	3	1 (3.7)	2 (8.3)	

IQR, interquartile range. Bold values indicate a statistically significant difference (*p* < 0.05)

## Discussion

In the present study, pelvic MRI and abdominal CT were analyzed in 136 patients with perianal fistulizing CD, and our results revealed differences in adipose tissue features among CD patients with different active perianal fistulas, that is, lower SAT and VAT density, higher SAI and VAI and lower VA/TA index correspond to lower perianal fistula activity, reflecting the relationship between perianal fistula and inflammatory load in CD patients.

CT body composition parameters have been used to reflect disease activity in CD patients and predict their prognosis. Feng et al. found that the *λ*_HU_ of creeping fat (CF) in CD patients increased with the severity of intestinal inflammation, and the *λ*_HU_ of CF around the intestinal segments without lesions was significantly higher than that in the controls [20]. Zhou et al. showed an increased risk of poor prognosis in CD patients with high SAT and VAT density [22]. Similarly, we observed higher VAT density at all levels in the high activity group (*p* < 0.01). Increased VAT density on CT images is often associated with elevated inflammation, suggesting increased inflammatory exudation in the diseased intestines. Therefore, VAT density is not only a distinguishing indicator of CD activity, but also reflects perianal fistula activity to some extent.

In Thiberge's study [21], CD patients with low SAI and VAI were at greater risk of adverse outcomes, which is supported by Zhou's results [22]. And the latter proposed standardizing SAT and VAT area with L1–L5 vertebral height instead of height to reduce reliance on clinical data, and the new standardized indexes obtained proved to be highly relevant to the traditional method. We adopted Zhou's standardized scheme and found lower SAI and VAI in the high activity group. However, some studies pointed out that high VAI in CD patients was associated with the occurrence of postoperative complications and increased risk of recurrence [28, 29], and they focused on demonstrating the role of VAT in the pathogenesis of CD. In fact, these indicators do not apply to patients with different nutritional status. Patients with longer disease duration tend to have lower contents of SAT and VAT as a result of disease consumption. But for some overweight or obese patients with a short course of disease, their SAT and VAT contents are generally higher than the former, but the disease activity is not necessarily lower. Therefore, if only the area or standardized indices are compared, the conclusions obtained tend to ignore their nutritional status.

In order to avoid the influence of absolute content parameters on conclusions, many studies have introduced relative content parameters Previous studies have shown that VSR or VA/TA index in CT images are higher in aggressive CD or patients with adverse outcomes [18, 22]; Buning et al. also found that high VAT/FM (total fat mass) ratio (the ratio of VAT to total body fat mass, similar to VA/TA index) was associated with more complex behaviors and higher activity by using MRI [30]. Moreover, in Bryant's study, in addition to finding higher VSR in patients with low life quality, they also found a positive correlation between VSR and stricturing disease behavior in ileocolonic CD patients [31]. We used the same parameters, and although the difference in VSR was not apparent between the two groups, higher VA/TA index at the L5 level was observed in the high activity group. It is also worth noting that patients in the high activity group had a higher percentage of stricturing disease behavior. Therefore, we believe that relative content indexes are more reliable when evaluating the negative impact of VAT on the course of CD. In addition, our analysis also shows a high correlation between the same parameters at the three lumbar levels. Therefore, researchers should consider possible differences at the L4 and L5 levels in addition to the L3 level commonly used in previous analyses when analyzing CD patients.

Previous studies mentioned that perianal disease can be the initial manifestation of CD, predating the onset of intestinal lesions [1, 32]. In our study, before being diagnosed with CD, forty-five patients underwent perianal fistula surgery, with a median time interval of two years, and the longest even reaching nine years. Usually, CD-induced perianal fistulas could not be cured with conventional treatment alone [33]. Patients with previous surgery in our study developed recurrent fistulas months or years later because they did not receive appropriate CD-related treatment. Therefore, it is necessary for clinicians to investigate the possibility of CD in patients who present with only perianal fistula. Even if patients do not show related gastrointestinal signs, they should be advised to pay attention to the occurrence of intestinal lesions in future routine physical examination.

Waheed’s study suggests that a complex or high perianal fistula on MRI images may be the initial presentation of CD, which differs from the general population [34]. By analyzing our patients, we found that 61.0% (83/136) presented with complex fistulas, whereas high fistulas were uncommon, only 8.1% (11/136). Oliveira et al. showed that the characteristics of perianal fistulas were actually similar in the CD and non-CD populations, as evidenced by abscess incidence, volume, and signs of fistula activity (high signal on T2WI images as well as focal enhancement); however, they also found that compared to the non-CD population, the CD population was younger (28.6 ± 14.9 y vs. 42.4 ± 14.7 y) and rectal inflammation was more common (30.2% vs. 6.7%) [35]. The mean age of our patients was 27.5 ± 10.2 years and the incidence of rectal involvement was 34.6% (47/136), similar to the CD population of Oliveira et al. (*p* = 0.51 and 0.49, respectively). Although we did not analyze the non-CD patients, our results support their conclusion that young patients with concomitant rectal inflammatory perianal fistulas are more likely to be further diagnosed with CD, which is associated with the involvement of the colorectum [2, 36].

Although no studies have verified consistency between perianal fistula activity and activity of intestinal or extraintestinal lesions in CD patients, it has been suggested that patients may have better outcomes after surgery when the intestinal disease is in a quiescent phase [37], reflecting the impact of systemic inflammation on perianal fistula treatment and prognosis. In the simple grouping we developed based on Van Assche's classification (low and high activity group), the high activity group had higher PDAI and laboratory inflammatory markers (CRP and ESR) and a higher proportion of complaints of perianal fistula-related symptoms (50.8% vs. 23.4%, *p* = 0.002), suggesting the validity of our grouping and revealing a correlation between perianal fistula activity and systemic activity in CD patients. Although we did not observe differences in CT parameters between patients with different outcomes, patients with low PDAI tended to be more responsive to treatment and had a better short-term prognosis in both low and high activity group, in line with the findings of Pikarsky et al. [38].

Our study has some limitations. Due to the retrospective design, the follow-up time and treatment were not controlled, and they did affect the outcome. Although the baseline results have not been affected, a rigorous prospective study should be carried to reveal the influence of CT parameters on the prognosis of perianal fistula.

In conclusion, we can simply divide perianal fistulas in CD patients into two states of high and low activity by using pelvic MRI, and fistula with high activity is related to higher VAT density and VA/TA index, suggesting higher overall inflammatory load.

## Supplementary Information

**Additional file 1:** Supplemental methods on semi-automatic segmentation, and supplemental figures and tables.

## Data Availability

The data underlying this article cannot be shared publicly due to the privacy of individuals that participated in the study. The data will be shared on reasonable request to the corresponding author.

## Abbreviations

CD: Crohn's disease
CRP: C-reactive protein
CT: Computed tomography
ESR: Erythrocyte sedimentation rate
HU: Hounsfield unit
MRI: Magnetic resonance imaging
PDAI: Perianal disease activity index
SAI: Subcutaneous adipose index
SAT: Subcutaneous adipose tissue
VAI: Visceral adipose index
VAT: Visceral adipose tissue
