# Supplementary material for: The relationship between perianal fistula activity and abdominal adipose tissue in Crohn’s disease: an observational study
Source: Insights Imaging. 2022 Sep 24;13:156. doi: 10.1186/s13244-022-01293-6 (PMC9509502; doi:10.1186/s13244-022-01293-6)
Supplement: Supplementary file 1 — Additional file 1: Supplemental methods on semi-automatic segmentation, and supplemental figures and tables. [file 13244_2022_1293_MOESM1_ESM.pdf]

ELECTRONIC SUPPLEMENTARY MATERIAL

The relationship between perianal fistula activity and abdominal adipose tissue in Crohn's disease: an observational study

Supplemental methods..2

Figure S1 A coronal CT image shows the measurement of vertebral height (heigh$_{L1-5}$)...........3

Figure S2 The selection of slices to be analyzed...3

Figure S3 Steps for outlining subcutaneous adipose tissue (SAT) and visceral adipose tissue (VAT) in abdominal CT images using ImageJ software..4

Table S1 Kappa coefficients of MRI evaluation results of two radiologists..............................5

Table S2 Comparison of CT body composition parameters between two groups.....................6

Table S3 Comparison of different follow-up outcomes in low activity group...........................7

Table S4 Comparison of different follow-up outcomes in high activity group..........................9

Supplemental methods

A research assistant selected 5 slices of images evenly within each patient's third to fifth three lumbar levels, for a total of 15 slices of images for subsequent analysis (Figure S2). Two trained radiologists outlined the subcutaneous adipose tissue (SAT) and visceral adipose tissue (VAT) in the above images using the semi-automated method of ImageJ (National Institutes of Health, https://imagej.nih.gov/ij/index.html). The process of adipose tissue segmentation is based on the tutorial of skeletal muscle segmentation by Gomez-Perez et al [Gomez-Perez S, McKeever L, Sheean P. Tutorial: A Step-by-Step Guide (Version 2.0) for Measuring Abdominal Circumference and Skeletal Muscle From a Single Cross-Sectional Computed-Tomography Image Using the National Institutes of Health ImageJ. *JPEN. Journal of parenteral and enteral nutrition*. 2020;44(3):419-424].

The main step was to display all adipose tissue by setting a threshold of -190 to -30HU, followed by manually erasing the intermuscular and intestinal adipose tissue to outline SAT and VAT, respectively.

The correction was carried out by another radiologist and consisted mainly of examination and correction of preliminary results. After completing the above steps, the area and density of SAT and VAT in each image were obtained by using the measure function of ImageJ, and then the average value of each vertebral level was calculated.

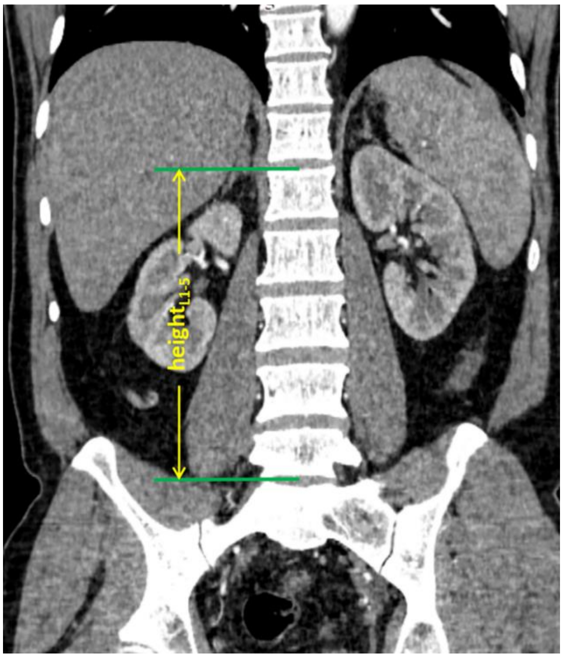

Figure S1 A coronal CT image shows the measurement of vertebral height ($heigh_{L1-5}$).

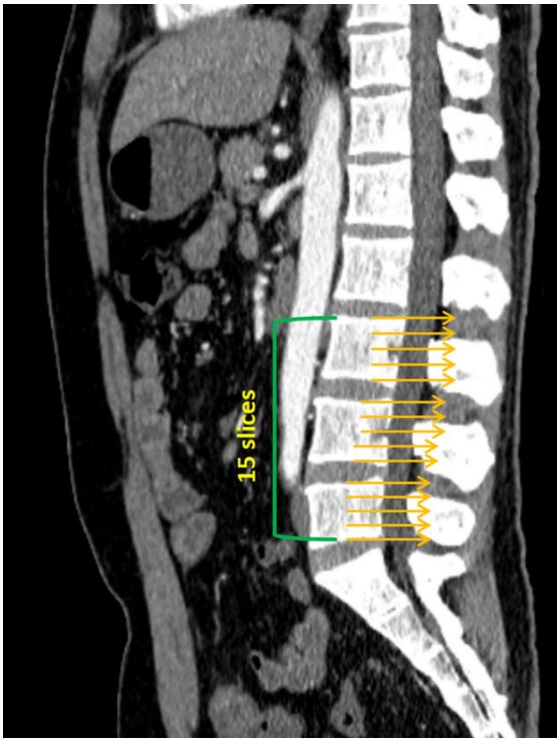

Figure S2 The selection of slices to be analyzed.

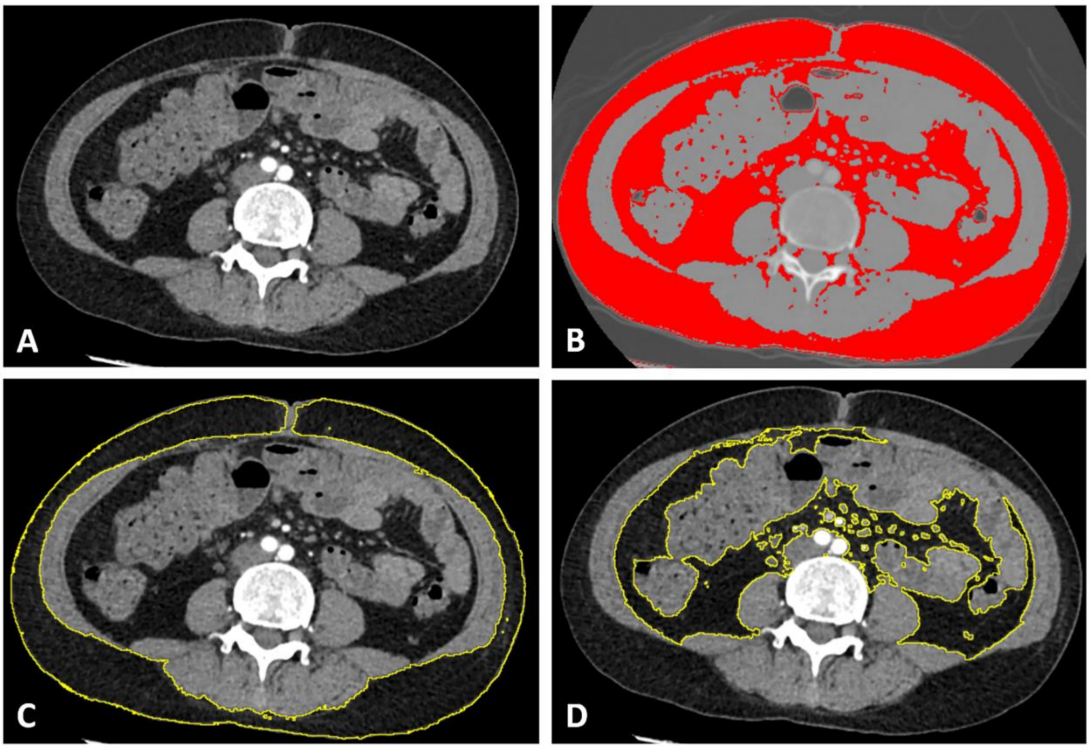

Figure S3 Steps for outlining subcutaneous adipose tissue (SAT) and visceral adipose tissue (VAT) in abdominal CT images using ImageJ software. **A:** One of a patient’s abdominal CT images; **B:** Display all adipose tissue by setting a threshold of -190 to -30HU, and then generate regions of interest (ROI); **C:** Erase adipose tissue except SAT to produce ROI of SAT; **D:** Erase adipose tissue except VAT to produce ROI of VAT.

Table S1 Kappa coefficients of MRI evaluation results of two radiologists

**Items**	**Kappa coefficient (95% CI)**
No. of fistula tracks	1.000
Location	0.985 (0.957 - 1.000)
Extension	1.000
Hyperintensity on T2WI	0.810 (0.682 - 0.938)
Collections (cavities＞3mm diameter)	1.000
Rectal wall involvement	1.000
Total score	0.893 (0.815 - 0.970)

CI, Confidence interval.

Table S2 Comparison of CT body composition parameters between two groups

	Total (n=136)	Low activity group (n=77)	High activity group (n=59)	P value
SAI, $\times10^{2-}$, cm^2/m^2				
L3 level	18.90±14.26	21.14±14.81	15.98±13.07	**0.015**
L4 level	22.85±16.40	25.33±16.83	19.61±15.37	**0.019**
L5 level	24.04±17.05	26.58±16.87	20.72±16.84	**0.017**
VAI, $\times10^{2}$, cm^2/m^2				
L3 level	16.70±12.14	19.03±12.64	13.67±10.83	**0.008**
L4 level	17.89±11.60	19.48±12.01	15.81±10.81	0.07
L5 level	17.20±9.59	18.38±10.24	15.67±8.52	0.17
VSR				
L3 level	1.14±1.46	1.03±0.53	1.29±2.14	0.70
L4 level	1.03±1.54	0.87±0.39	1.24±2.29	0.36
L5 level	1.04±1.58	0.83±0.43	1.31±2.33	0.07
VA/TA index				
L3 level	0.48±0.12	0.48±0.11	0.48±0.14	0.94
L4 level	0.45±0.12	0.45±0.11	0.47±0.13	0.28
L5 level	0.45±0.13	0.43±0.11	0.48±0.14	**0.04**
SAT density, HU				
L3 level	-81.78±22.97	-87.74±21.74	-74.01±22.38	<**0.001**
L4 level	-84.06±22.83	-90.02±21.30	-76.29±22.58	<**0.001**
L5 level	-80.51±23.63	-86.93±21.80	-72.13±23.47	<**0.001**
VAT density, HU				
L3 level	-69.29±14.77	-72.48±15.13	-65.11±13.29	**0.004**
L4 level	-69.28±15.01	-72.88±15.39	-64.58±13.21	**0.001**
L5 level	-67.44±14.48	-71.10±14.59	-62.65±12.96	<**0.001**

SAI, subcutaneous adipose index; VAI, visceral adipose index; VSR, VAT/SAT ratio; VA/TA: VAT/Total adipose tissue; SAT, subcutaneous adipose tissue; VAT, visceral adipose tissue.

Table S3 Comparison of different follow-up outcomes in low activity group

	Responders (n=13)	Non-responders (n=14)	P value
**Baseline data**			
Age	28.54±10.24	26.93±11.10	0.50
Sex			0.68
Male	9	11	
Female	4	3	
C-reaction protein, mg/L	27.92±19.73	37.34±39.29	0.83
ESR, mm/hr	21.69±13.94	30.08±24.82 (n=13)	0.30
PDAI (mean±SD)	2.1±0.3	2.6±0.76	**0.04**
Van Assche (mean±SD)	11.0±2.2	11.6±1.9	0.50
Disease behaviour			0.29
B1	8	9	
B2	3	1	
B3	1	4	
B2+3	1	0	
SAI, ×10^{-3}, cm^2/m^2			
L3 level	18.71±10.59	22.51±19.55	0.72
L4 level	22.32±10.85	27.22±22.82	0.91
L5 level	23.68±10.94	28.01±22.67	0.94
VAI, ×10^{-3}, cm^2/m^2			
L3 level	16.09±10.13	17.94±14.87	0.94
L4 level	16.19±8.06	19.37±14.81	0.98
L5 level	16.77±7.14	17.81±11.29	0.91
VSR			
L3 level	0.94±0.42	0.90±0.44	0.38
L4 level	0.80±0.37	0.79±0.34	0.91
L5 level	0.77±0.24	0.82±0.45	0.79
VA/TA index			
L3 level	0.46±0.13	0.45±0.10	0.84
L4 level	0.42±0.11	0.43±0.10	0.97
L5 level	0.43±0.08	0.42±0.12	0.97
SAT density, HU			
L3 level	-90.14±20.50	-82.91±25.33	0.43
L4 level	-92.39±20.43	-85.48±24.27	0.94
L5 level	-89.51±19.59	-81.14±25.98	0.72
VAT density, HU			
L3 level	-71.66±13.18	-66.85±17.25	0.43
L4 level	-73.16±12.47	-68.73±17.50	0.46

L5 level	-71.93±12.38	-67.12±16.05	0.39
**Follow-up data**			
Time interval, months	6 (3.5, 9.5)	5.5 (3.75, 24.75)	
Therapy			0.10
Drugs	12	10	
Surgery	-	-	
Drugs+surgery	1	-	
Untreated	-	4	

ESR, Erythrocyte sedimentation rate; PDAI, Perianal Disease Activity Index; SAI, subcutaneous adipose index; VAI, visceral adipose index; VSR, VAT/SAT ratio; VA/TA: VAT/Total adipose tissue; SAT, subcutaneous adipose tissue; VAT, visceral adipose tissue.

Table S4 Comparison of different follow-up outcomes in high activity group

	Reduced to low activity (n=12)	Remained high activity (n=12)	P value
**Baseline data**			
Age	25.92±12.40	23.33±9.64	0.58
Sex			1.00
Male	10	9	
Female	2	3	
C-reaction protein, mg/L	53.89±44.67	52.94±35.65	0.96
ESR, mm/hr	32.20±17.22	39.27±20.03	0.40
PDAI (mean±SD)	2.9±2.1	9.8±5.4	**0.002**
Van Assche (mean±SD)	17.9±2.5	18.3±1.8	0.65
Disease behaviour			1.00
B1	9	9	
B2	2	3	
B3	1	-	
SAI, $\times 10^2$, cm^2/m^2			
L3 level	13.67±10.70	17.42±13.35	0.46
L4 level	16.72±12.83	20.78±14.56	0.48
L5 level	17.80±14.23	20.89±14.92	0.61
VAI, $\times 10^2$, cm^2/m^2			
L3 level	12.55±14.13	14.34±12.73	0.75
L4 level	14.40±13.54	15.83±11.38	0.78
L5 level	15.15±10.61	15.04±8.48	0.98
VSR			
L3 level	0.93±0.42	0.96±0.76	0.90
L4 level	0.88±0.30	0.85±0.56	0.89
L5 level	1.03±0.43	0.87±0.56	0.45
VA/TA index			
L3 level	0.46±0.11	0.43±0.16	0.66
L4 level	0.45±0.09	0.42±0.14	0.51
L5 level	0.49±0.10	0.43±0.13	0.25
SAT density, HU			
L3 level	-71.06±21.50	-78.96±22.02	0.38
L4 level	-72.31±21.66	-82.18±20.61	0.27
L5 level	-66.54±23.95	-78.15±20.83	0.22
VAT density, HU			
L3 level	-64.52±12.42	-66.43±16.19	0.75
L4 level	-63.29±11.92	-66.30±16.45	0.61
L5 level	-62.79±11.33	-64.39±16.21	0.78
**Follow-up data**			
Time interval, months	11 (4, 22)	6 (3.25,13.5)	0.25

Therapy			**0.004**
Drugs	10	2	
Surgery	-	3	
Drugs+surgery	1	5	
Untreated	1	2	

ESR, Erythrocyte sedimentation rate; PDAI, Perianal Disease Activity Index; SAI, subcutaneous adipose index; VAI, visceral adipose index; VSR, VAT/SAT ratio; VA/TA: VAT/Total adipose tissue; SAT, subcutaneous adipose tissue; VAT, visceral adipose tissue.
